# Use of Congo red dye-formaldehyde as a new sensitizer-reductant couple for enhanced simultaneous solar energy conversion and storage by photogalvanic cells at the low and artificial sun intensity

**DOI:** 10.1038/s41598-020-76388-5

**Published:** 2020-11-06

**Authors:** Pooran Koli, Yashodhara Dayma, Ramesh Kumar Pareek, Meenakshi Jonwal

**Affiliations:** Department of Chemistry, Jai Narain Vyas University, Jodhpur, Rajasthan 342001 India

**Keywords:** Chemistry, Energy science and technology, Engineering, Materials science, Optics and photonics

## Abstract

The photogalvanic cells (PG) are the promising and renewable electrochemical energy devices capable of doing the simultaneous solar power generation and storage. To realize the aim of the practical application of the PG cells in daily life, the electrical output of these cells has to be further enhanced to a level at least comparable to that of the photovoltaic cells. The present study of the PG cells based on so far unexplored Congo red dye-formaldehyde as a photosensitizer-reductant couple along with efficiency enhancer surfactant reagent (sodium lauryl sulfate) in the sodium hydroxide alkaline medium has shown greatly enhanced cell performance over published results. The present study has shown electrical cell performance of the PG cell as P_pp_ 782 μW, i_sc_ 3200 μA, V_oc_ 1074 mV, and CE 11.02% at artificial and low illumination intensity. The storage capacity (t_0.5_) of the PG cell has been observed in the present study as 120 min in the dark. The study of variation of the different cell fabrication parameters has shown optimum cell performance at an optimal value of these cell fabrication parameters. The most plausible mechanism of the photo-generation of the current in PG cells is also proposed on the basis of observed potential values and published literature.

## Introduction

The solar energy coming from the sun is an everlasting renewable source of the energy. Solar power has the potential to become a reliable energy source as a supplement and substitute to the limited and polluting fossils’ fuels. The solar techniques like photovoltaic cells (PV), photogalvanic cells (PG)^1^, DSSCs^2–4^, etc., have ability to convert the solar energy directly into the solar electrical energy. The PG cells are quite different from the other solar cell techniques cells like PV cells. The PG cells are only solar cells which have inherent solar power storage capacity with stored power retrievable in the dark^5^. The PG cell works on the principle of the photogalvanic effect in which the photochemical changes occurring in the solution causing photopotential generation on illumination. Rideal and Williams were the first scientists to observe the photogalvanic effect^6^, and the Rabinowitch was the first researcher who not only systematically investigated this effect, but also gave the idea that this effect can be used for the solar energy conversion and storage^7,8^. To exploit this idea of the Rabinowitch, the PG cells have been extensively studied for solar energy conversion and storage by using two electrodes (anode and cathode) dipped in the solution having photosensitizer, reductant, NaOH, and surfactant (surfactant in some cases)^9–12^. The electrical performance (current, power, potential, efficiency, etc.) of the PG cells have been reported as dependent on the cell fabrication parameters like concentrations, diffusion length, temperature, electrode nature, Pt electrode size, illumination intensity, etc. Therefore, the various researchers have focused mainly on these cell fabrication parameters for updating and enhancing the electrical performance of PG cell technology. Various dye sensitizers, organic reductants, and micelles have been studied to increase the electrical performance of the PG cells. The chemical combination of the sensitizer-reductant-surfactant-NaOH may be labeled as a photogalvanic chemical system. The various photogalvanic chemical systems like the azur A (a dye photosensitizer)-glycerol (organic reductant)-sodium lauryl sulphate (NaLS, an anionic surfactant)-NaOH (as alkaline medium)^13^; azur C (a dye photosensitizer)-glycerol (organic reductant)-Triton X 100 (a nonionic surfactant)-NaOH (as alkaline medium)^14^; the azur A (a dye photosensitizer)- ethylenediaminetetraacetic acid (EDTA, an organic reductant)-NaLS (an anionic surfactant)-NaOH (as alkaline medium)^15^; the brilliant cresyl (a dye photosensitizer)-oxalic acid (an organic reductant)-NaOH (as alkaline medium)^16^; the congo red (a dye photosensitizer)- D-Xylose (an organic reductant)- cetyl pyridinium chloride (CPC, a cationic surfactant)-NaOH (as alkaline medium)^17^, the safranine O (a dye photosensitizer)-EDTA (an organic reductant)- NaLS (an anionic surfactant)-NaOH (as alkaline medium)^18^, the Sudan I (a dye photosensitizer)-Fructose (an organic reductant)-NaLS (an anionic surfactant)-NaOH (as alkaline medium)^19^, the Fast Green FCF (a dye photosensitizer)-Fructose (an organic reductant)- NaLS (an anionic surfactant)-NaOH (as alkaline medium)^20^, etc. have been studied in PG cells for the simultaneous solar energy conversion and storage. The photogalvanic chemical system based on mixed dyes photosensitizer (Brilliant Cresylblue + Toluidine Blue)-ethylene glycol (an organic reductant)- NaLS (an anionic surfactant) has also been exploited for enhanced photogalvanics^21^. The studied so far described have focused mainly on the use of reference saturated calomel electrode (SCE) and large size anodic Pt electrode (1 cm × 1 cm) leading to the relatively low electrical cell performance of the PG cell. The PG cells are the diffusion controlled cells depending on the diffusion of the ions in the bulk of the electrolytic solution. Therefore, the diffusion and sensitivity of the electrodes is one of the main determinants of their efficiency. So to ensure good diffusion and sensitivity, the small size Pt electrode (which creates less hindrance to the mobility of the ions) with more sensitive combination electrode (only SCE terminal used) has attracted the attention of the researchers to successfully improve the PG cell performance to a high level as 649.6 μW power (P_pp_), 2250 μA current (i_sc_), 1048 mV potential (V_oc_), 8.12% conversion efficiency (CE), and 59 min power storage capacity (as half change time, t_0.5_) at artificial and low illumination intensity. To realize the aim of practical application of the PG cells in daily life, the electrical output of these cells has to be further enhanced to a level at least comparable to that of the PV cells. The literature survey reveals that the dye aggregation retards the dye diffusion leading to the reduced PG cell performance. The dye decay is also reported as an adverse factor for dye sensitizer based PG cells. Both these factors (dye aggregation and dye decay) are reported to be tackled by the use of micelles (anionic micelles like NaLS more effective), and high pH alkaline medium. The Congo red dye is also reported to show decreased aggregation at high pH, and this property of Congo red makes it fit for good photogalvanics. The formaldehyde has two hydrogen atoms attached to the carbonyl group, and this peculiar position makes its hydrogen atoms easily oxidisable making it (formaldehyde) a good candidate as a reducing agent (standard reduction potential 0.237 V vs SHE). So in totality, the position emerges out of the literature survey is that the use of small size Pt electrode, combination electrode, high pH range and NaLS surfactant in alkaline medium with so far unexplored congo red dye-formaldehyde system could be a route for further updating and enhancing the electrical performance of the PG cells, and therefore, the present study was undertaken.

The present study of the PG cells based on so far unexplored Congo red dye-formaldehyde as photosensitizer-reductant couple along with efficiency enhancer surfactant reagent (sodium lauryl sulphate) in sodium hydroxide alkaline medium has shown greatly enhanced cell performance over published results. The present study has shown electrical performance of the PG cell as P_pp_ 782 μW, i_sc_ 3200 μA, V_oc_ 1074 mV, and CE 11.02% at artificial and low illumination intensity. The storage capacity (t_0.5_) of the PG cell has been observed in present study as 120 min in the dark. The study of variation of the different cell fabrication parameters has shown optimum cell performance at an optimal value of these cell fabrication parameters. The most plausible mechanism of the photo-generation of the current in PG cells is also proposed on the basis of observed potential values and published literature. The novelty and originality of the present work lies in the fact that the use of unexplored Congo red dye photosensitizer-formaldehyde reductant couple has shown abruptly enhanced results over published work.

## Materials and methods

### Materials used

The chemicals like Congo red dye, Sodium Lauryl Sulfate, Formaldehyde, Sodium Hydroxide, Oxalic acid, Phenolphthalein, and singly distilled water have been used as photosensitizer, surfactant, reductant, alkaline medium, standard reagent for standardization of alkali NaOH, indicator, and solvent for preparing solutions, respectively. All solutions were stored in the amber colored vessels with the aim of protecting them from the sunlight induced decay (see Section 1 of Supplementary Information-SI).

### Method

**Figure 1:**
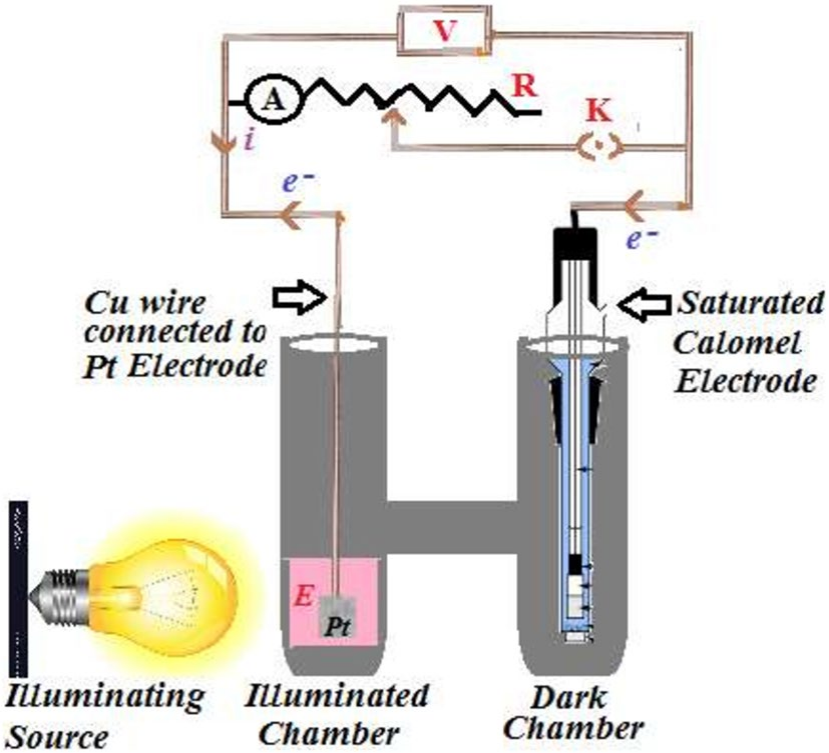
Photogalvanic experimental setup (A, Micro-ammeter; K, Key; R, A typical single-turn potentiometer (acts as a variable resistor or rheostat); V, digital pH meter; e^−^, electron; i, current; E, electrolytic solution having dye sensitizer, surfactant, reductant, NaOH; Pt as working electrode; Saturated Calomel electrode as counter electrode).

The stock solutions of all chemicals were prepared at different concentrations in the singly distilled water and kept away from the sun. The known amount of the solutions of the Congo red dye photosensitizer, formaldehyde reductant, sodium lauryl sulfate surfactant, and standardized alkali sodium hydroxide was filled in the H-shaped glass tube (diffusion length, 7.5 cm). The total volume of the solution was kept 68 ml making up by the singly distilled water. The H-shaped glass tube was externally blackened leaving a small illuminating window in a limb (called illuminated chamber). Then platinum electrode of size (0.5 cm × 0.3 cm) was dipped in the illuminated limb and combination electrode was immersed in another limb of the H-cell. The terminals of the electrodes were connected to a digital pH meter, micro ammeter, carbon pot (log 470 K), and a key to make a complete circuit. The H-cell was put in the dark to attain a stable potential (called dark potential-V_dark_). Then, through the illuminating window, the solution near the platinum electrode was illuminated with the artificial sunlight coming from the incandescent Tungsten lamp of 200 Wattage. The photo potential and photo current were measured with help of digital pH meter and micro ammeter, respectively. During the experiment, variables i (current) and V (voltage) were observed. The plot i *v*_*s*_ V gave information about efficiency of the storage of energy and conversion of stored solar energy into electrical energy. The experimental setup of photogalvanic cell is given in Fig. 1^9,22^.

In literature, various formulas are reported for the calculation of the solar energy conversion efficiency of the photogalvanic cells. Potter and Thaller^23^ have defined the power efficiency as [(½ E^0^_c_ I_c_)/(ϵN)] , where E^0^_c_ is zero current voltage, I_c_ is the short circuit current, Є is the energy per absorbed photon and N is the number of photons absorbed per second. Clark and Eckert^24^ have defined Sunlight Engineering Efficiency as [(maximum Power × 100)/(incident sunlight power)]. Murthy et al.^25^ have defined conversion efficiency as [(V_oc_ × i_sc_ × fill factor × 100)/(cross sectional area of electrode × Incident Sunlight Power)]. A review paper on photogalvanic cells have reported calculation of conversion efficiency as [(electrical power output (in watts) at its maximum power point × 100)/(input light in Wm^−2^ × electrode area of the photogalvanic cell in m^2^)]^26^. The fill factor of photogalvanic cells is disproportionately low in comparison to conversion efficiency. Both the parameters (fill factor and efficiency) are indicator of solar energy conversion capacity of the photogalvanic cells. Therefore, the solar efficiency of cell as without linked to fill factor and linked to fill factor has also been defined as [(power at power point in m Wm^−2^ × 100%)/(illuminating intensity in m Wm^−2^ × Pt working electrode area in cm^2^)], and [(power at power point in m Wm^−2^ × fill factor × 100%)/(illuminating intensity in m Wm^−2^ × Pt working electrode area in cm^2^)], respectively^9,11^. In present research work, the cell efficiency has been calculated as [(power at power point in m Wm^−2^ × fill factor × 100%)/(illuminating intensity in m Wm^−2^ × Pt working electrode area in cm^2^)]. The justification of using this formula is explained as follows. The size of the open window has not been used for calculating the efficiency as—(1) the sunlight strike at illuminating window of the cell. The sunlight being of wave nature has phenomenon of scattering as well the diffraction at hole/barrier. The photogalvanic cells use very dilute solution of dye/pigments. The light diffraction effect and edge effect effectively illuminate the larger solution area, (2) the PG cell is diffusion controlled, and therefore, only those dye molecules which are nearer to Pt anodic electrode shall be able to reach the electrode within their excited life time. It means that the number of such dye molecules shall be determinable with respect to Pt area, but not the illuminating area of the cell window size; (3) the illuminating natural sunlight is free and available naturally leading to no change in cost and environmental pollution on account of illumination of the small or large area of the cell. The Pt size matters for the economic and ecological cost as use of its small size will be less costly and more eco-friendly; (4) The Pt anode area (instead of cell illumination area) for calculation of the efficiency is also supported by Murthy et al*.*^25^, who have used cross sectional area of the anode for the calculation of solar engineering efficiency; and (5) The photogalvanic cells can also be charged at low illuminating efficiency^9^.Therefore, the small or large sized illuminating window hardly matters for charging of the cell. The surface area of the window used to illuminate the cell is 1 cm × 1 cm.

## Mechanism of photocurrent, photo-potential generation and storage capacity

The photogalvanic cell has two chambers. One is illuminated chamber having platinum electrode (anode) and other is dark chamber having saturated calomel electrode (cathode).

*Illumination chamber *The dye molecules get excited and excited dye molecules accept an electron from the reductant and get converted into its semi or leuco form-

1 $$ {\text{Dye}} + {\text{h}}\nu \left( {\text{light photon}} \right) \to {\text{Dye }}\left( {\text{S}} \right)* \to {\text{Dye }}\left( {\text{T}} \right)* $$

2 $$ {\text{Dye }}\left( {\text{T}} \right)* \, + {\text{ R}} \to {\text{Dye}}^{-} \left( {\text{semi or leuco}} \right) \, + {\text{ R}}^{ + } $$

*At platinum electrode *The semi or leuco form of the dye molecules loses an electron and gets converted into original molecules.

3 $$ {\text{Dye}}^{{{-} }} \to {\text{Dye }} + {\text{ e}}^{-} $$

*Dark chamber* At Saturated Calomel Electrode, the dye molecules accept an electron and get converted into semi form.

4 $$ {\text{Dye}} + {\text{e}}^{-} \to {\text{Dye}}^{-} \left( {{\text{semi}}} \right) $$

Finally, semi form of dye and oxidized form of reductant combine to give original dye and reductant molecule in dark chamber. The cycle continues goes on in cell.

5 $$ {\text{Dye}}^{-} + {\text{ R}}^{ + } \to {\text{Dye}} + {\text{R}} $$

Here Dye, Dye*, Dye^–^, R and R^+^ are the ground state dye, excited form of dye, semi or leuco form of dye, reductant and its oxidized form, respectively.

## Results and discussion

### Study of the Congo red dye photosensitizer in photogalvanic system with formaldehyde as reductant

The present study of the use of Congo red dye as photo sensitizer, Formaldehyde as Reductant, Sodium lauryl sulphate as surfactant, Sodium hydroxide as alkaline medium and small Pt electrode (0.5 cm × 0.3 cm) at an artificial light has been done in H-shape glass tube vessel for further improvement in the electrical output of the cell. The cell showed the results as open circuit-potential, short circuit current, conversion efficiency, fill factor, and power at power points as 1074 mV, 3200 µA, 11.02%, 0.22, and 782 µW, respectively. The storage capacity of the photogalvanic cell was obtained 120 min. in the dark. The effect of different parameters on the electrical output of the cell was observed as described below.

### Effect of the variation of the dye (Congo red) concentration in Congo red-formaldehyde photogalvanic system

The effect of variation of the dye sensitizer concentration on Congo red-Formaldehyde system has been studied by fabricating the five photogalvanic cells. The concentrations of the formaldehyde reductant, SLS surfactant, NaOH alkali (i.e. pH), diffusion length, Pt electrode size and SCE are same for all the five cells, but the concentration of Congo red dye sensitizer is different in different cells (See sec. 2 of SI).

Table 1 shows the highest electrical output for the [dye] = 1.0 × 10^–4^ M. Therefore, the 1.0 × 10^–4^ M dye concentration is the optimal dye concentration for this photogalvanic system. The optimal cell performance is V_oc_ = 1074 mV, i_max_ = 6000 µA, i_sc_ = 3200 µA, P_pp_ = 782 μW, FF = 0.22, CE = 11.02%, and charging time ‘t’ = 35 min.

**Table 1:** Effect of the variation of the Congo red dye photo-sensitizer concentration.

Electric parameters of PG cell	[Congo red dye concentration] × 10^–4^ M				
	0.7	0.8	1.0	1.1	1.3
V_oc_ (mV)	1011	1028	1074	1039	1010
t (min)	15	15	35	20	40
i_max_ (µA)	5000	3500	6000	4500	5000
i_sc_ (μA)	1600	2200	3200	2900	1700
P_pp_ (μW)	170	528	782	649	162
CE (%)	5.72	0.67	11.02	11.64	0.93
FF	0.07	0.02	0.22	0.28	0.09

At [Formaldehyde] = 1.4 × 10^–3^ M, [SLS] = 1.0 × 10^−2^ M, Pt electrode area = 0.5 cm × 0.3 cm, Light intensity = 10.4 mW cm^−2^, Diffusion length (D_L_) = 7.5 cm, pH = 13.82.

On increasing the concentration of the Congo red dye photo-sensitizer, there is an increase in the photocurrent, and the optimum cell performance is observed at 1.0 × 10^–4^ M concentration of the Congo red dye (Table 1). This pattern of the change of photocurrent with the Congo red dye photo-sensitizer may be attributed to the fact that at a lower concentration range of the Congo red dye photo-sensitizer there will be limited numbers of the photo-sensitizer molecules to absorb the photons and to donate the electrons to the Pt electrode in the cell. The higher concentration of the Congo red dye photo-sensitizer will not permit the desired light intensity to reach the dye photo-sensitizer molecules near the electrode and hence there will be a corresponding fall in the power of the cell.

### Effect of the variation of the reductant (formaldehyde) concentration on Congo red-formaldehyde photogalvanic system

The effect of variation of formaldehyde reductant concentration on Congo red-Formaldehyde photogalvanic system has been studied by fabricating the five photogalvanic cells. The concentrations of Congo red dye photo-sensitizer, SLS surfactant, NaOH alkali (i.e. pH), diffusion length, Pt electrode size and SCE are same for all five cells, but the Formaldehyde concentration is different in different cells (see sec. 3 of SI).

Table 2 shows the highest electrical output of the cell for [Formaldehyde] = 1.4 × 10^–4^ M. Therefore, the 1.4 × 10^–4^ M formaldehyde concentration is the optimal Formaldehyde concentration for this photogalvanic system. The optimal cell performance is V_oc_ = 1074 mV, i_max_ = 6000 µA, i_sc_ = 3200 µA, P_pp_ = 782 μW, FF = 0.22, CE = 11.02%, and charging time ‘t’ = 35 min.

**Table 2:** Effect of the variation of the Formaldehyde reductant concentration.

Electric parameters of PG cell	[Formaldehyde concentration] × 10^–3^ M				
	1.1	1.3	1.4	1.5	1.6
V_oc_ (mV)	991	1048	1074	1016	1012
t (min)	25	25	40	55	15
i_max_ (µA)	4000	5000	6000	4500	4500
i_sc_ (μA)	1100	1100	3200	1500	1400
P_pp_ (μW)	143	174	782	197	188
CE (%)	1.18	1.65	11.02	1.51	1.56
FF	0.13	0.15	0.22	0.12	0.13

At [Dye] = 1.0 × 10^−4^ M, [SLS] = 1.2 × 10^−2^ M, Pt electrode area = 0.5 cm × 0.3 cm, Light intensity = 10.4 mW cm^−2^, Diffusion length (D_L_) = 7.5 cm, pH = 13.82.

On increasing the concentration of the Formaldehyde reductant at the constant value of other cell fabrication variables, the electrical output of the PG cell is found to increase to reach a maximum and optimum value, and thereafter, it is found to decrease. The optimum cell performance is observed at 1.4 × 10^–3^ M concentration of the Formaldehyde reductant (Table 2). The electrical output is low at a lower concentration range of the Formaldehyde reductant as there will be its lesser number of the molecules to donate electrons to the excited molecules of the Congo red dye photo-sensitizer. The higher concentration of the formaldehyde reductant may hinder the movement of the Congo red dye molecules towards the electrodes in the desired time limit, and may also promote back electron transfer from the Congo red dye molecule to Formaldehyde reductant molecule.

### Effect of the variation of NaOH concentration (pH) on the Congo red-formaldehyde photogalvanic system

The effect of variation of the NaOH concentration (i.e., pH) on Congo red-Formaldehyde photogalvanic system has been studied by fabricating the five photogalvanic cells. The concentrations (of Formaldehyde reductant, Congo red dye sensitizer, and SLS surfactant), diffusion length, Pt electrode size and SCE are same for all five cells, but the NaOH (i.e. pH) concentration is different in different cells (See sec.4 of SI).

Table 3 shows the highest electrical output of the cell for pH = 13.82. Therefore, the 13.82 is the optimum pH for this photogalvanic system. The optimal cell performance is V_oc_ = 1074 mV, i_max_ = 6000 µA, i_sc_ = 3200 µA, P_pp_ = 782 μW, FF = 0.22, CE = 11.02%, and charging time ‘*t*’ = 35 min.

**Table 3:** Effect of the NaOH concentrations on the system.

Electrical parameters of PG cell	pH				
	13.77	13.79	13.82	13.83	13.85
V_oc_ (mV)	1058	1070	1074	1057	1031
t (min)	15	20	35	20	20
i_max_ (µA)	5000	5000	6000	5000	3000
i_sc_ (μA)	2800	3000	3200	1600	1500
P_pp_ (μW)	728	763	782	398	353
CE (%)	11.19	11.24	11.02	5.86	9.3
FF	0.24	0.23	0.22	0.23	0.22

At [Dye] = 1.0 × 10^–4^ M, [Formaldehyde] = 1.4 × 10^–3^ M, [SLS] = 1.0 × 10^–2^ M, Pt electrode area = 0.5 cm × 0.3 cm, Light intensity = 10.4 mW cm^−2^, Diffusion length (D_L_) = 7.5 cm.

It is observed that the PG cell system works effectively in the strong alkaline medium. The photocurrent of the cell is high in higher pH range. The maximum photocurrent and power at power point has been observed at an optimum pH 13.82 (Table 3). On further increasing the pH, there is a decrease in the photocurrent, and it may be due to the fact that at the very high concentration of the OH^−^ (i.e., pH), the OH^−^ may combine with the oxidized state the formic acid reductant inhibiting regeneration of its original state.

### Effect of the variation of the surfactant (SLS) on Congo red-formaldehyde photogalvanic system

The effect of the variation of SLS surfactant concentration on the Congo red-Formaldehyde photogalvanic system has been studied by fabricating the five photogalvanic cells. The concentrations [of the Congo red dye sensitizer, HCHO, NaOH (i.e. pH)], diffusion length, Pt electrode size and SCE are same for all five cells, but the SLS surfactant concentration is different in different cells (See sec. 5 of SI).

Table 4 shows the highest electrical output of the cell for [SLS] = 1.0 × 10^–2^ M. Therefore, the [SLS] = 1.0 × 10^−2^ M is the optimum concentration of SLS for this photogalvanic system. The optimal cell performance is V_oc_ = 1074 mV, i_max_ = 6000 µA, i_sc_ = 3200 µA, P_pp_ = 782 μW, FF = 0.22, CE = 11.02%, and charging time ‘t’ = 35 min.

**Table 4:** Effect of concentrations of the SLS surfactant on the system.

Electrical parameter of the PG cell	[SLS] × 10^−2^ M				
	9.5	1.0	1.1	1.2	1.3
V_oc_ (mV)	1049	1074	1032	1131	1013
t (min)	45	35	40	25	20
i_max_ (µA)	5000	6000	5000	4500	5000
i_sc_ (μA)	2000	3200	1600	1700	1600
P_pp_ (μW)	427	782	182	176	162
CE (%)	5.47	11.02	1.28	0.11	0.09
FF	0.20	0.22	0.11	0.09	0.93

At [Dye] = 1.0 × 10^–4^ M, [Formaldehyde] = 1.4 × 10^–3^ M, Pt electrode area = 0.5 cm × 0.3 cm, Light intensity = 10.4 mW cm^−2^, Diffusion length (D_L_) = 7.5 cm, pH = 13.82.

On increasing the concentration of SLS at the constant value of all other cell fabrication variables, the electrical output of the PG cell is found to increase to reach a maximum and optimum value. The optimum cell performance is observed at 1.0 × 10^–2^ M concentration of the SLS surfactant (Table 4). At SLS surfactant concentration lower than the 1.0 × 10^–2^ M concentration, the lower value of the electrical output may be attributed to the lesser number of SLS surfactant molecules available for the electron transfer and the solubility of the Congo red dye photo-sensitizer molecules. The higher concentration of the SLS surfactant may be hindering the motion of Congo red dye photo-sensitizer molecules toward the electrodes leading to the corresponding fall in the power of the PG cell. Further, the SLS surfactant also is able to give electrons to the dye by the charge transfer process enhancing the cell performance. The SLS also reduces the back electron transfer reaction by making surrounding micelles structure about the sensitizer molecule leading to the enhanced cell performance.

### Variation of the potential, current, and power with the Platinum electrode area in the Congo red-formaldehyde system based photogalvanic cell

Photogalvanic cells uses Pt electrode as working electrode. An exploratory study has shown dependence of the photo-generated current, potential and power on the Pt electrode size (See sec.6 of SI). For electrodes of area larger than this, the cell parameters were found decreasing with increase in electrode area (Table 5). For the observed effect of electrode area, the better cell parameters were found for small electrodes owing to relatively less hindrance to diffusion of ions as the Photogalvanic cells are based on ion diffusion mechanism. This observation is in agreement with the other dye sensitizer (other than Congo red) also^9^. The efficiency calculation is based on Pt electrode size. The main electrical parameter of the cell is the power generated to decide the number of solar panels (solar cells) to be used for establishing the solar plant capacity (KW, or MW or GW) for meeting energy needs of a locality. As far as the power generated is concerned, the electrode area 0.50 cm × 0.30 cm has shown highest power in the experimental conditions. Under the observed effect of electrode area, the i_max_, i_sc_, and P_pp_ were found highest for electrode area 0.50 cm × 0.30 cm. It is the reason that the whole study in present research has been done with Pt of electrode area 0.50 cm × 0.30 cm. It is an optimized area under the research conditions. Below this area, the Pt area will be small and so it will provide surface for lesser number of electron rich sensitizer molecules for dye-Pt interaction leading to reduce current and power generation.

**Table 5:** Variation of the potential, current, and power with the Platinum electrode area in the Congo red-formaldehyde system based photogalvanic cell.

Electrical parameter of the PG cell	Pt electrode area (cm^2^)			
	0.40 × 0.20	0.50 × 0.30	0.50 × 0.50	1.00 × 1.00
V_oc_ (mV)	1049	1074	1095	1013
t (min)	25	35	39	45
i_max_ (µA)	4500	6000	4400	4000
i_sc_ (μA)	2900	3200	2400	1000
P_pp_ (μW)	649	782	590	170

At [Dye] = 1.0 × 10^–4^ M, [Formaldehyde] = 1.4 × 10^–3^ M,[SLS] = 1.2 × 10^−2^ M, Light intensity = 10.4 mW cm^−2^, Diffusion length (D_L_) = 7.5 cm, pH = 13.82.

### Variation of the potential, current, and power in the Congo red-formaldehyde system based photogalvanic cell

The variation of the potential during charging of the cell is shown in Table S1, and Fig. S1. This Fig. S1 shows the maximum potential V_max_ = 1080 mV, and open-circuit potential V_oc_ = 1074 mV. The change in the potential and current was observed during the charging of the cells. On illuminating each photogalvanic cell, the potential increases regularly and reaches to a highest value (V_max_), which then decrease and becomes quite constant (V_oc_) after some time (Table S1, and Fig. S1).

**Figure 2:**
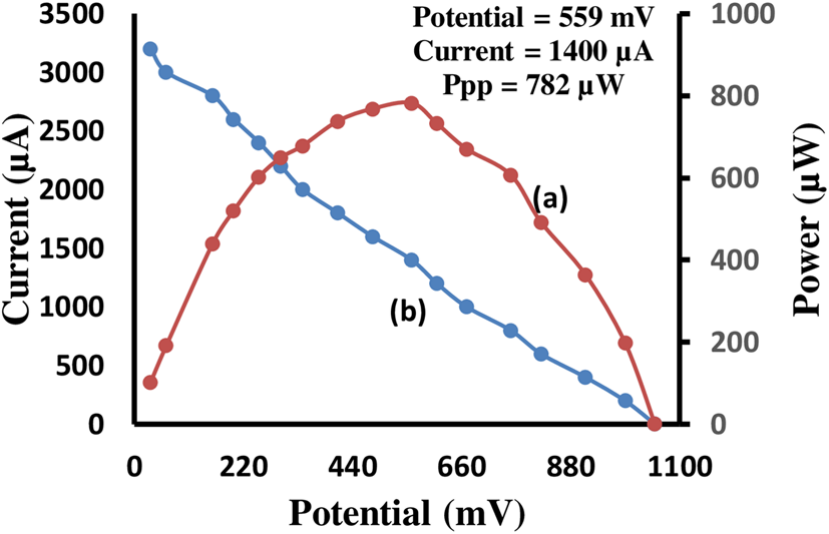
Variation of the power and current with the potential, (a) Power versus Potential (b) i–V characteristics of the cell (where, ‘a’ curve between primary ‘X’ axis and secondary ‘Y’ axis, and ‘b’ curve between primary ‘X’ axis and primary ‘Y’ axis).

**Figure 3:**
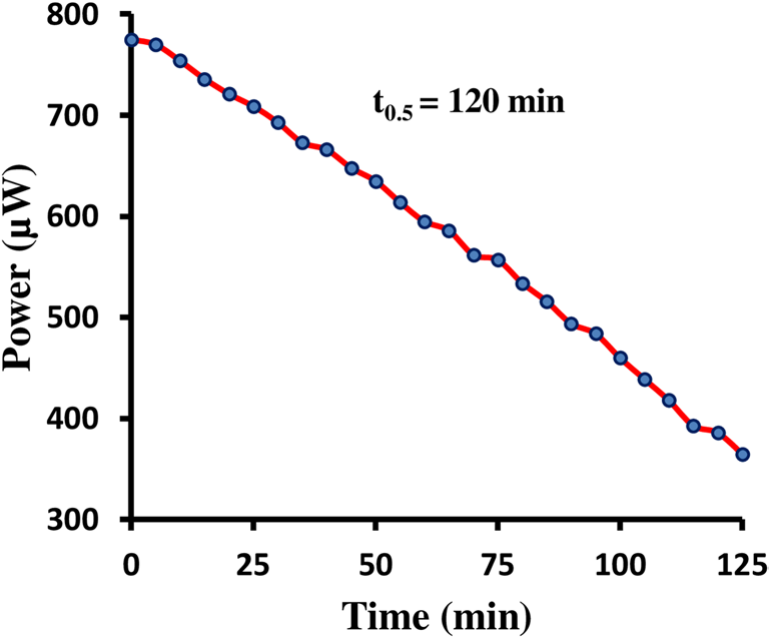
Storage capacity of the cell (retrieval of the stored power in dark from the cell).

With the help of the digital pH meter and micro ammeter, we observed the short circuit current (i_sc_) and open circuit voltage (V_oc_) respectively of the cell. The current and potential values in between these two extreme values were recorded with the help of carbon plot connected in the circuit of micro ammeter, through which an external load was applied. The current–voltage (i–V) characteristics of the cell were reported in Table S2 and graphically represented in the Fig. 2 (variation of the potential and power with current). The storage capacity of the photo galvanic cell system containing the Congo red was determined by applying the desired external load with keeping the cell at the power point stage in dark and noted down the time required in fall of power output to its half value. The storage capacity of the photo galvanic cell system was denoted by t_1/2_ (half change time), which was the measure of the performance of the cell. It was observed that the cell could be used in dark for 120 min (Table 6, Fig. 3).

**Table 6:** Storage capacity of the cell (retrieval of the stored power in dark from the cell).

Time (min)	Potential (mV)	current (µA)	Power (µW)
0	1400	554	775
10	1400	539	754
20	1400	515	721
30	1400	495	693
40	1400	476	666
50	1400	454	635
60	1400	425	595
70	1400	402	562
80	1400	382	534
90	1400	354	494
100	1400	329	460
110	1400	299	418
120	1400	276	386

The i–V characteristic of the cell is shown in the Table S2, and Fig. 2. The Fig. 2 shows that potential increases with decreases in the current. The current and power data and curve is also shown in Table S2, and Fig. 2. The maxima of this curve show the power at power point of the cell and in this system it is 782 µW. And, the current at power point (i_pp_) and potential at power point (V_pp_) for this photogalvanic system is 1400 µA and 559 mV, respectively.

The cell performance in the dark is recorded in the Table 6, and Fig. 3. It is clear from the Fig. 3 that the power decreases with time in dark during retrieval of the stored power from the cell. Initially, the power is P_pp_ (782 µW), and after 120 min the power is 391 µW. This duration of the 120 min is half change time (t_0.5_) of this photogalvanic system. The half change time is defined as the time in which the power reduces in dark during retrieval of the stored power to half of its initial value.

The optimum cell performance at values of the cell variable is summarized as dark potential (V_dark_) 692 mV; maximum potential (V_max_) 1080 mV; open-circuit potential (V_oc_) 1074 mV; photo potential (∆V) 388 mV; charging time (t) 35 min; maximum current (i_max_) 6000 µA; short-circuit current (i_sc_) 3200 µA; power at power point (P_pp_) 782 µW; potential at power point (v_pp_) 559 mV; current at power point (i_pp_) 1400 µA; t_0.5_ 120 min, potential at t_0.5_ (V_0.5_) 276 mV; current at t_0.5_ (i_sc_) 1400 µA; conversion efficiency (CE) 11.02%; fill factor (FF) 0.22.

The results in present work are impressive and an improvement in the field of photogalvanics. This is explicit from the recently published work on Congo red dye. The i_sc_ 110.0 µA, P_pp_ 45.0 µW, FF 0.74, CE 0.66%, and t_0.5_ 40 min were reported for Congo red dye-Glycerol system (with Pt 1.0 cm × 1.0 cm)^22^. The i_sc_ 470 µA, P_pp_ 119.6 µW, FF 0.2356, CE 1.1500%, and t_0.5_ 130 min were reported for Congo red dye-D-Xylose-Cetyl pyridinium chloride system (with Pt 1.0 cm × 1.0 cm)^27^. The i_sc_ 180 µA, P_pp_ 32.46 µW, CE 1.04%, and t_0.5_ 120 min were reported for Congo red dye-EDTA system (with Pt 1.0 cm × 1.0 cm)^28^. The i_sc_ 972 µA, P_pp_ 244.02 µW, CE 7.58%, and t_0.5_ 3.6 h were reported for Fast Green FCF dye with Fructose as reductant in NaOH as alkaline medium (with Pt 1.0 cm × 1.0 cm)^29^. The i_sc_ 45 µA, P_pp_ 14.75 µW, CE 0.14%, and t_0.5_ 40 min were reported for Azur B dye-EDTA reductant-Tergitol-7 surfactant system (with Pt 1.0 cm × 1.0 cm)^30^. The latest published work has reported electrical output of these cells as high as P_pp_ 649.6 μW, i_sc_ 2250 μA, V_oc_ 1048 mV, CE 8.12%, and t_0.5_ 59 min at artificial and low illumination intensity.

The literature survey reveals that the dye aggregation retards the dye diffusion leading to the reduced PG cell performance. The dye decay is also reported as an adverse factor for dye sensitizer based PG cells. Both these factors (dye aggregation and dye decay) are reported to be tackled by the use of micelles (anionic micelles like NaLS, which is more effective), and high pH alkaline medium. The Congo red dye is also reported to show decreased aggregation at high pH and low concentration (dye)^31^, and this property of Congo red makes it fit for good photogalvanics. The formaldehyde has two hydrogen atoms attached to the carbonyl group, and this peculiar position makes its hydrogen atoms easily oxidisable making it (formaldehyde) a good candidate as a reducing agent (standard reduction potential 0.237 V vs SHE). Thus, the advancement in results in the present work may be attributed to factors like the use of anionic surfactant, higher pH, small Pt electrode, combination electrode, formaldehyde reductant, Congo red dye sensitizer, very low concentration of Congo red dye, etc. Present Congo red-Formaldehyde-NaLS systems have used anionic surfactant (i.e., NaLS) as micelles. Congo red-Glycerol system^22^ has not used any surfactant, and Congo red dye-D-Xylose-Cetyl pyridinium chloride system^27^ has not used anionic, but cationic surfactant.In previous works, the size of Pt electrode was large (1.0 cm × 1.0 cm), but no one used small size Pt electrode and combination electrode. Present work has used both small size Pt (0.5 cm × 0.3 cm) electrode and combination electrode. Both (anionic surfactant and small Pt electrode) help in enhancing the diffusion due to which cell performance also increases. Small Pt electrode creates less hindrance for ion diffusion in the cell. Thus, the dye Congo red coupled with small Pt electrode gives more current and power. Similarly, the anionic surfactant provides for good solubility and electron injection capacity of the dye. It facilitates and increases rate of transfer of electron to Pt electrode, which in turn gives more current and power.

The increase in solubility of cationic Congo red dye in the presence of surfactants is also supported from the published work. Shahir et al. have reported the detection of formation of dye-surfactant ion pairs, their small mixed aggregates (below the critical micelle concentration (CMC) of these surfactants) and surfactant micelles using the conductometry, spectroscopy, tensionmetry, and pulsed field gradient NMR (PFG-NMR) technology. Above the CMC, the dye is reported to be reverted to its monomeric state and solubilized in the micelles. A hydrophobic interaction as well as electrostatics is reported to contribute to the dye/surfactant interactions^32^.

The present research of photogalvanics based on Congo red dye photosensitier-formaldehyde reductant- surfactant reagent (sodium lauryl sulfate) has yielded promising results in terms of the power and efficiency. This photogalvanics of Congo red dye is prone to face similar challenges as are faced by the other dye based photogalvanics. Malviya and Solanki have listed some challenges as back thermal reactions (electron goes back from anodic electrode to leuco/semi form of dye), photochemical side reactions (the photo-chemical degradation competes with photogalvanics), electrode aging (electrode corrosion diminishes the activity of electrodes), low conversion efficiency, etc.^26^.

For future research, the use of various measures is suggested to overcome these limitations. The dye solubility, diffusion and electrode selectivity with fast electron transfer kinetics is the key factors affecting the cell efficiency and dye-anodic electrode interaction. Higher the diffusion will favor this interaction and in turn photogalvanics over photo-chemical reactions of excited dye molecules. A Vertical Architecture provides for increasing the efficiency of the photogalvanic solar cell. In vertical illumination, light enters the cell to move through the area between two electrodes, that is different from the conventional approach in which the cell is illuminated through the electrode. The vertical illumination causes spreading of the light absorption and electron generation through the depth of the device ensuring absorption of all the incoming photons even with low dye solubility. In effect, the spreading of electron generation facilitates increased solar energy conversion even at slow electrode kinetics^33^.

Highly efficient novel scandium supported TiO_2_ HOMBIKAT UV 100 (Sc/TiO_2_) working electrode^34^ and ZnO nanostructure working electrode are suggested for overcoming electrode related limitations of the photogalvanics^35^.

The surfactant plays a key role in the solubility and stability of the dye molecules. Regarding this, the choice for a most suitable surfactant chemical may be made from the work of Tiwari et al.^36^. The surfactant for which the dye-surfactant interaction affects critical micellar concentration more effectively can be most suitable micelles for enhancing the overall electrical performance of the photogalvanic cells^36^.

## Conclusion

Sensitizer-reductant couple is one of the main determinants for deciding the performance of the photogalvanic cells. The decreased aggregation of the dye coupled with good reductant favour the efficiency of these cells. The Congo red dye is reported to show decreased aggregation at high pH, and this property of Congo red makes it fit for good photogalvanics. The formaldehyde has two hydrogen atoms attached to the carbonyl group, and this peculiar position makes its hydrogen atoms easily oxidisable making it (formaldehyde) a good candidate as a reducing agent (standard reduction potential 0.237 V vs SHE). So in totality, the hypothesis emerged out was that the use of so far unexplored congo red dye-formaldehyde system along with small size Pt electrode, combination electrode, high pH range and NaLS surfactant in alkaline medium could be a route for further updating and enhancing the electrical performance of the PG cells. The present study has shown greatly enhanced cell performance over published results. The present study has shown electrical performance of the PG cell as P_pp_ 782 μW, i_sc_ 3200 μA, V_oc_ 1074 mV, and CE 11.02% at artificial and low illumination intensity. The storage capacity (t_0.5_) of the PG cell has been observed in present study as 120 min in the dark. The study of variation of the different cell fabrication parameters has shown optimum cell performance at an optimal value of these cell fabrication parameters.

## Data availability

The data that supports the findings of this study are available within the article.

## Supplementary information

Supplementary Information.
