# Supplementary material for: Use of Congo red dye-formaldehyde as a new sensitizer-reductant couple for enhanced simultaneous solar energy conversion and storage by photogalvanic cells at the low and artificial sun intensity
Source: Sci Rep. 2020 Nov 6;10:19264. doi: 10.1038/s41598-020-76388-5 (PMC7648078; doi:10.1038/s41598-020-76388-5)
Supplement: Supplementary file 1 — Supplementary Information. [file 41598_2020_76388_MOESM1_ESM.docx]

**Supplementary Information for**

Use of Congo red dye-formaldehyde as a new sensitizer-reductant couple for enhanced simultaneous solar energy conversion and storage by photogalvanic cells at the low and artificial sun intensity

Pooran Koli*, Yashodhara Dayma, Ramesh Kumar Pareek, and Meenakshi Jonwal, Department of Chemistry, Jai Narain Vyas University, Jodhpur -342001, Rajasthan, India

**Corresponding Author, poorankoli@yahoo.com;* [*poorankoli@rediffmail.com*](mailto:poorankoli@rediffmail.com)

1. **Material used**

The chemicals like Congo red dye (a acidic azo group dye, 85 % Assay Purity, C_32_ H_22_ N_6_ Na_2_ O_6_ S_2_, MW 696.68, ʎ_max_ 497.0 nm, Loba Chemie), Sodium Lauryl Sulfate (NaC_12_H_25_SO_4_, MW 288.38, Sisco Research Laboratories), Formaldehyde (37.41 % Assay, HCHO, MW 30.03 g mol^-1^, Ases Chemicals), Sodium Hydroxide (NaOH, Minimum assay 98 % Assay, MW 40.0 g mol^-1^, Ases Chemicals), Oxalic acid (C_2_H_2_O_4_, MW 90.034 g mol^-1^), and singly distilled water have been used for cell fabrication.

The solutions 0.002 M Congo red dye, 0.1M Sodium Lauryl Sulfate, 0.01M Formaldehyde, 1M Sodium Hydroxide, 1 M Oxalic acid, Phenolphthalein, and singly distilled water have been used as photosensitizer, surfactant, reductant, alkaline medium, standard reagent for standardization of alkali NaOH, indicator, and solvent for preparing solutions, respectively. All solutions were stored in the amber colored vessels with the aim of protecting them from the sunlight induced decay.

The appliances like digital pH meter Systronics modal 335, micro-ammeter OSAWA, H-shaped glass tube, 200 W incandescent tungsten lamp, saturated calomel electrode (SCE) component of the combination electrode, Pt electrode, carbon pot log 470 K device (potentiometer), and a circuit key were used for measuring potential, for measuring current, for accommodating the electrolytic solution and two electrodes dipped in this solution, as illumination source, as reference electrode and positive terminal of the PG cell, as negative terminal of the PG cell, for varying the resistance of circuit, and for closing and opening the circuit, respectively.

1. **Effect of the variation of the dye (Congo red) concentration in Congo red-formaldehyde photogalvanic system**

For fabricating a cell, the 45 ml of 1M NaOH, 7 ml of M/10 SLS, and 10 ml of M/100 HCHO has been taken. Along with this amount of the solutions of NaOH, SLS, and HCHO; the 2.5 ml of M/500 Congo red dye solution + 3.5 ml of single distilled water was taken in the 1^st^ cell; the 3 ml of M/500 Congo red dye solution + 3 ml of single distilled water was taken in the 2^nd^ cell; the 3.5 ml of M/500 Congo red dye solution + 2.5 ml of single distilled water was taken in the 3^rd^ cell; the 4 ml of M/500 Congo red dye solution + 2 ml of single distilled water was taken in the 4^th^ Cell; and the 4.5 ml of M/500 Congo red dye solution + 1.5 ml of single distilled water was taken in the 5^th^ cell. In each cell, the total volume of the solution including water was 68 ml. For 1^st^ cell, the cell fabrication parameters were resultant concentrations as [dye] = 0.7 × 10^-4^ M, [Formaldehyde] = 1.4 × 10^-3^ M, [SLS] = 1.0 × 10^-2^ M, pH = 13.82; Pt electrode area = 0.5 cm × 0.3 cm, Light intensity = 10.4 mWcm^-2^, and Diffusion length (D_L_) = 7.5 cm. For 2^nd^ cell, the cell fabrication parameters were resultant concentrations as [dye] = 0.8 × 10^-4^ M, [Formaldehyde] = 1.4 × 10^-3^ M, [SLS] = 1.0 × 10^-2^ M, pH = 13.82; Pt electrode area = 0.5 cm × 0.3 cm, Light intensity = 10.4 mWcm^-2^, and Diffusion length (D_L_) = 7.5 cm. For 3^rd^ cell, the cell fabrication parameters were resultant concentrations as [dye] = 1.0 × 10^-4^ M, [Formaldehyde] = 1.4 × 10^-3^ M, [SLS] = 1.0 × 10^-2^ M, pH = 13.82; Pt electrode area = 0.5 cm × 0.3 cm, Light intensity = 10.4 mWcm^-2^, and Diffusion length (D_L_) = 7.5 cm. For 4^th^ cell, the cell fabrication parameters were resultant concentrations as [dye] = 1.1 × 10^-4^ M, [Formaldehyde] = 1.4 × 10^-3^ M, [SLS] = 1.0 × 10^-2^ M, pH = 13.82; Pt electrode area = 0.5 cm × 0.3 cm, Light intensity = 10.4 mWcm^-2^, and Diffusion length (D_L_) = 7.5 cm. And for the 5^th^ cell, the cell fabrication parameters were resultant concentrations as [dye] = 1.3 × 10^-4^ M, [Formaldehyde] = 1.4 × 10^-3^ M, [SLS] = 1.0 × 10^-2^ M, pH = 13.82; Pt electrode area = 0.5 cm × 0.3 cm, Light intensity = 10.4 mWcm^-2^, and Diffusion length (D_L_) = 7.5 cm.

1. Effect of the variation of the reductant (Formaldehyde) concentration on Congo red-Formaldehyde photogalvanic system

For fabricating a cell, the 45 ml of 1M NaOH, 3.5 ml of M/500 dye, and 7 ml of M/10 SLS has been taken. Along with this amount of solutions of the NaOH, dye, and SLS; the 8 ml of M/100 Formaldehyde solution + 4.5 ml of single distilled water was taken in the 1^st^ cell; the 9 ml of M/100 Formaldehyde solution + 3.5 ml of single distilled water was taken in the 2^nd^ cell; the 10 ml of M/100 Formaldehyde solution + 2.5 ml of single distilled water was taken in the 3^rd^ cell; the 10.5 ml of M/100 Formaldehyde solution + 2 ml of single distilled water was taken in the 4^th^ cell; and the 11 ml of M/100 Formaldehyde solution + 1.5 ml of single distilled water was taken in the 5^th^ cell. In each cell, the total volume of the solution including the water was 68 ml.

For 1^st^ cell, the cell fabrication parameters were resultant concentrations as [dye] = 1.0 × 10^-4^ M, [Formaldehyde] = 1.1 × 10^-3^ M, [SLS] = 1.0 × 10^-2^ M, pH = 13.82; Pt electrode area = 0.5 cm × 0.3 cm, Light intensity = 10.4 mWcm^-2^, and Diffusion length (D_L_) = 7.5 cm. For 2^nd^ cell, the cell fabrication parameters were resultant concentrations as [dye] =1.0 × 10^-4^ M, [Formaldehyde] = 1.3 × 10^-3^ M, [SLS] = 1.0 × 10^-2^ M, pH = 13.82; Pt electrode area = 0.5 cm × 0.3 cm, Light intensity = 10.4 mWcm^-2^, and Diffusion length (D_L_) = 7.5 cm. For 3^rd^ cell, the cell fabrication parameters were resultant concentrations as [dye] = 1.0 × 10^-4^ M, [Formaldehyde] = 1.4 × 10^-3^ M, [SLS] = 1.0 × 10^-2^ M, pH = 13.82; Pt electrode area= 0.5 cm × 0.3 cm, Light intensity = 10.4 mWcm^-2^, and Diffusion length (D_L_) = 7.5 cm. For 4^th^ cell, the cell fabrication parameters were resultant concentrations as [dye] =1.0 × 10^-4^ M, [Formaldehyde] = 1.5 × 10^-3^ M, [SLS] = 1.0 × 10^-2^ M, pH = 13.82; Pt electrode area = 0.5 cm × 0.3 cm, Light intensity = 10.4 mWcm^-2^, and Diffusion length (D_L_) = 7.5 cm. And, for the 5^th^ cell, the cell fabrication parameters were resultant concentrations as [dye] =1.0 × 10^-4^ M, [Formaldehyde] = 1.6 × 10^-3^ M, [SLS] = 1.0 × 10^-2^ M, pH = 13.82; Pt electrode area = 0.5 cm × 0.3 cm, Light intensity = 10.4 mWcm^-2^, and Diffusion length (D_L_) = 7.5 cm.

1. Effect of the variation of NaOH concentration (pH) on the Congo red-formaldehyde photogalvanic system

For fabricating a cell, the 3.5 ml of M/500 ml dye, 7 ml of M/10 SLS, and 10 ml of M/100 HCHO has been taken. Along with this amount of solutions of dye, SLS, and HCHO; the 38 ml of 1M NaOH solution + 9.5 ml single distilled water was taken in the 1^st^ cell; the 40 ml of 1 M NaOH solution + 7.5 ml of single distilled water was taken in the 2^nd^ cell; the 42 ml of 1 M of NaOH solution + 5.5 ml of single distilled water was taken in the 3^rd^ cell; the 45 ml of 1M NaOH solution + 2.5 ml of single distilled water was taken in the 4^th^ cell; and the 46 ml of 1M NaOH solution + 1.5 ml of single distilled water was taken in the 5^th^ cell. In each cell, the total volume of solution including the water was 68 ml.

For 1^st^ cell, the cell fabrication parameters were resultant concentrations as [dye] = 1.0 × 10^-4^ M, [Formaldehyde] = 1.4 × 10^-3^ M, [SLS] = 1.0 × 10^-2^ M, pH = 12.75; Pt electrode area = 0.5 cm × 0.3 cm, Light intensity = 10.4 mWcm^-2^, and Diffusion length (D_L_) = 7.5 cm. For 2^nd^ cell, the cell fabrication parameters were resultant concentrations as [dye] =1.0 × 10^-4^ M, [Formaldehyde] = 1.4 × 10^-3^M, [SLS] = 1.0 × 10^-2^ M, pH = 13.77; Pt electrode area = 0.5 cm × 0.3 cm, Light intensity = 10.4 mWcm^-2^, and Diffusion length (D_L_) = 7.5 cm. For 3^rd^ cell, the cell fabrication parameters were resultant concentrations as [dye] =1.0 × 10^-4^ M, [Formaldehyde] = 1.4 × 10^-3^ M, [SLS] = 1.0 × 10^-2^ M, pH = 13.79; Pt electrode area = 0.5 cm × 0.3 cm, Light intensity = 10.4 mWcm^-2^, and Diffusion length (D_L_) = 7.5 cm. For 4^th^ cell, the cell fabrication parameters were resultant concentrations as [dye] =1.0 × 10^-4^ M, [Formaldehyde] = 1.4 × 10^-3^ M, [SLS] = 1.0 × 10^-2^M, pH = 13.82; Pt electrode area = 0.5 cm × 0.3 cm, Light intensity = 10.4 mWcm^-2^, and Diffusion length (D_L_) = 7.5 cm. And, for the 5^th^ cell, the cell fabrication parameters were resultant concentrations as [dye] = 1.0 ×10^-4^ M, [Formaldehyde] = 1.4 × 10^-3^ M, [SLS] = 1.0 × 10^-2^ M, pH = 13.83; Pt electrode area = 0.5 cm × 0.3 cm, Light intensity = 10.4 mWcm^-2^, and Diffusion length (D_L_) = 7.5 cm.

1. Effect of the variation of the surfactant (SLS) on Congo red-Formaldehyde photogalvanic system

For fabricating a cell, the 45 ml of 1M NaOH, 3.5 ml of M/500 dye, and 10 ml of M/100 HCHO has been taken. Along with this amount of the solutions of NaOH, dye, and HCHO; the 6.5 ml of M/10 SLS solution + 3 ml of single distilled water was taken in the 1^st^ cell; the 7 ml of M/100 Formaldehyde solution + 2.5 ml of single distilled water was taken in the 2^nd^ cell; the 7.5 ml of M/100 Formaldehyde solution + 2 ml single of single distilled water was taken in the 3^rd^ cell; the 8 ml of M/100 Formaldehyde solution + 1.5 ml of single distilled water was taken in the 4^th^ cell; and the 8.5 ml of M/100 Formaldehyde solution + 1 ml of single distilled water was taken in the 5^th^ cell. In each cell, the total volume of solution including the water was 68 ml. For 1^st^ cell, the cell fabrication parameters were resultant concentrations as [dye] = 1.0 × 10^-4^ M, [Formaldehyde] = 1.4 × 10^-3^M, [SLS] = 0.95 × 10^-2^ M, pH = 13.82; Pt electrode area = 0.5 cm × 0.3 cm, Light intensity = 10.4 mWcm^-2^, and Diffusion length (D_L_) = 7.5 cm. For 2^nd^ cell, the cell fabrication parameters were resultant concentrations as [dye] = 1.0 ×10^-4^ M, [Formaldehyde] = 1.4 × 10^-3^ M, [SLS] = 1.0 × 10^-2^ M, pH = 13.82; Pt electrode area = 0.5 cm × 0.3 cm, Light intensity = 10.4 mWcm^-2^, and Diffusion length (D_L_) = 7.5 cm. For 3^rd^ cell, the cell fabrication parameters were resultant concentrations as [dye] =1.0 × 10^-4^ M, [Formaldehyde] = 1.4 ×10^-3^ M, [SLS] = 1.1 × 10^-2^ M, pH = 13.82; Pt electrode area = 0.5 cm × 0.3 cm, Light intensity = 10.4 mWcm^-2^, and Diffusion length (D_L_) = 7.5 cm. For 4^th^ cell, the cell fabrication parameters were resultant concentrations as [dye] =1.0 × 10^-4^ M, [Formaldehyde] = 1.4 ×10^-3^ M, [SLS] = 1.2 × 10^-2^M, pH = 13.82; Pt electrode area = 0.5 cm × 0.3 cm, Light intensity = 10.4 mWcm^-2^, and Diffusion length (D_L_) = 7.5 cm. And, for the 5^th^ cell, the cell fabrication parameters were resultant concentrations as [dye] =1.0 × 10^-4^ M, [Formaldehyde] = 1.4 ×10^-3^ M, [SLS] = 1.3 ×10^-2^M, pH = 13.82; Pt electrode area = 0.5 cm × 0.3 cm, Light intensity = 10.4 mWcm^-2^, and Diffusion length (D_L_) = 7.5 cm.

6. Variation of the potential, current, and power with the Platinum electrode area in the Congo red-formaldehyde system based photogalvanic cell

An exploratory study on the variation of the potential, current, and power with the Platinum electrode area in the Congo red-formaldehyde system has been done by fabricating four photogalvanic cells.

For fabricating each cell, the 45 ml of 1M NaOH, 3.5 ml of M/500 dye, 7 ml of M/10 SLS, 10 ml of M/100 HCHO, and 2.5 ml of single distilled water has been taken. In each cell, the total volume of solution including the water was 68 ml. For each cell, the cell fabrication parameters were resultant concentrations as [dye] = 1.0 × 10^-4^ M, [Formaldehyde] = 1.4 × 10^-3^ M, [SLS] = 1.2 × 10^-2^ M, pH = 13.82; Light intensity = 10.4 mWcm^-2^, and Diffusion length (D_L_) = 7.5 cm. For 1^st^, 2^nd^, 3^rd^, and 4^th^ cells, the Pt electrode area was 0.4 cm × 0.2 cm, 0.5 cm × 0.3 cm, 0.5 cm × 0.5 cm, and 1.0 cm × 1.0 cm, respectively.

Table S1. Variation of the potential with time during charging the cell.

| Time (min.) | 0 | 5 | 10 | 15 | 20 | 25 | 30 | 35 |
| --- | --- | --- | --- | --- | --- | --- | --- | --- |
| Potential (mV) | 692 | 824 | 1001 | 1008 | 1024 | 1034 | 1080 (V_max_) | 1074 (V_oc_) |

Table S2. Variation of the potential and power with the current (i-V characteristics of cell)

Current (µA) Potential^*^ (mV) Power (µW)

3200 32 102

3000 64 192

2800 157 439

2600 200 520

2400 251 602

2200 295 649

2000 339 678

1800 410 738

1600 480 768

1400 559 782

1200 610 732

1000 670 670

800 759 607

600 820 492

400 910 364

200 990 198

0 1050 0

*^*^Negative value*

**Fig.S1.** The performance of the cell (variation of the potential during charging of the cell).
